# Responsiveness to cold snaps by turtle embryos depends on exposure timing and duration

**DOI:** 10.1098/rspb.2024.2445

**Published:** 2025-01-15

**Authors:** Clinton R. Warren, Anthony T. Breitenbach, Rachel M. Bowden, Ryan T. Paitz

**Affiliations:** ^1^ School of Biological Sciences, Illinois State University, Normal, IL 61790, USA

**Keywords:** *Cyp19a1*, *Dmrt1*, *Kdm6b*, sex ratio, temperature-dependent sex determination

## Abstract

Characterizing how organisms respond to transient temperatures may further our understanding of their susceptibility to climate change. Past studies in the freshwater turtle, *Trachemys scripta*, have demonstrated that the timing and duration of heat waves can have major implications for the response of genes involved in gonadal development and the production of female hatchlings. Yet, no study has considered how the response of these genes to transient cold snap exposure may affect gonadal development and the production of males. We investigated how cold snap timing affects gonadal gene expression in *T. scripta* embryos and how the duration of an early cold snap influences the resulting hatchling sex ratios. Results show that responsiveness to cold changes rapidly across development, such that genes that responded when exposure began on incubation day 14 responded differently when exposure occurred just four or eight days later. Sex ratio data revealed that embryos experiencing an early cold snap also require a long exposure (>20 days) before most commit to testis development, suggesting that warm baseline temperatures may lower their sensitivity to later cold snap exposures. These results highlight how individual responses to incubation temperature can change rapidly across development in turtles and have important effects on sex ratios.

## 1. Introduction

As climate change progresses, organisms across the globe are anticipated to experience increasing bouts of unusual and extreme temperatures. Most notably, the rapid rise in average global temperatures, and the rising frequency and severity of transient heat waves [1], are likely to influence the physiology, ecology and life history of various organisms. Given the speed at which climate change is causing these anomalous temperatures, it is of conservation interest to identify which organisms are most vulnerable, or which might be less likely to adapt quickly enough to prevent severe population declines or extinction [2–4]. Among vertebrates, oviparous species may be especially vulnerable given that their embryonic development is highly contingent upon the environmental conditions they experience [5–11]. Although predicting the effects of climate change on development is difficult owing to the complicated interactions between environmental variables, it is generally suspected that the future rise in incubation temperatures will be largely maladaptive for many oviparous vertebrates [12–14]. For example, anomalously high temperatures are associated with nest failure in bird species that build open cup nests [15], and in lizards, thermal spikes increase embryonic mortality and alter the physiology of embryos that do survive to hatch [16]. While plastic and evolutionary adaptive capacity in embryos or parents may alleviate some selective pressure [4,17,18], the unprecedented rate of warming places many oviparous species at risk [19–23].

Turtles with temperature-dependent sex determination (TSD) may have additional fitness consequences associated with exposures to unusually warm or cool temperatures during embryonic development. In species with TSD, bipotential gonads respond to temperatures above or below a certain threshold, termed the pivotal temperature, by differentiating into either testes or ovaries [24–26]. Although not exclusive to turtles, TSD is found in nearly all Chelonians, including all sea turtles, and many freshwater turtles and tortoises [27]. In most turtles, warmer temperatures promote ovary development while cooler temperatures promote testis development [28]. As such, it is believed that most turtle populations are at risk of becoming increasingly female-biased under projected climate change models unless plasticity or evolutionary adaptation counteracts the effects of rising temperatures [29,30]. Developmental traits, such as TSD, are of special concern when it comes to the future viability of turtle populations owing to the potential for severe sex bias to cause reduced breeding capacity and genetic diversity [31]. Indeed, some turtle populations may already be experiencing severe female bias owing to global warming ([32,33]; but see [34–37]). This, when coupled with the typical physiological consequences of temperature extremes and the slow reproductive maturity of many species [38,39], often places turtles in the spotlight of conservation concern.

Given the potential consequences of climate change for species with TSD, a lot of thought has been given to how turtles may adapt to the changing climate [14], with a great deal of focus on predicting their responses to warming and transient heat waves. Adaptive responses may include changes in the reproductive behaviours of adults and/or the thermal sensitivity of embryos. Nesting behaviour is considered a trait that may be capable of mediating a response sooner than genetic or physiological responses [40]. However, while advancement in nesting date may be a means by which oviparous species reduce exposures to high temperatures during embryonic development ([29,30,41,42]; but see [43]), these shifts could also result in increased exposures to bouts of cool temperatures that might negatively impact fitness, survival or other biological responses. For example, New York populations of the tree swallow (*Tachycineta bicolor*) have seen decreased fitness and increased mortality in chicks resulting from increased exposures to cold snaps owing to progressively earlier nesting times ([44,45]; but see [46]). It therefore stands to reason that similar phenological responses in turtles could also cause increased exposure to cool temperatures. Similarly, longer summers resulting from climate change may extend the nesting date in some turtles and increase their production of late-season clutches [47–49], which might in turn be more likely to experience transient cold temperatures late in embryonic development as summer transitions to autumn. Importantly, climate change may also increase the severity and frequency of not only heat waves but also transient cold snaps [1]. Despite these potential ways in which turtles may experience increased exposures to transient cold temperatures during development, less attention is being paid to their embryonic responses to cool temperatures as compared to the focus on warm temperatures.

In addition to phenological shifts in response to climate change, another putative adaptative response is to lower sensitivity to warm temperatures and/or increase sensitivity to cool temperatures to offset warming in the future. Therefore, it may be important to understand how embryos of oviparous species respond to transient cool temperatures irrespective of nesting phenology. This is especially pertinent for species with TSD, where relatively short temperature exposures can result in irreversible commitment to either testes or ovaries [50]. It was recently demonstrated in the red-eared slider (*Trachemys scripta*) that embryos incubating at 27 ± 3°C hatch after 66 days and are 100% male, while embryos that are shifted to 29.5 ± 3°C for just 9 days hatch after 64 days but are only 33% male [51]. These findings illustrate how a short exposure to warm temperatures can trigger ovary differentiation, yet we know very little about how short exposures to cool temperatures influence testis differentiation and resulting sex ratios. It is also important to note that there is variation in how individuals respond to transient temperatures. For example, while some individuals appeared to be highly responsive and developed ovaries after just 5 days of heat exposure, others appeared to have low responsiveness and instead developed testes even after 15 days of heat exposure [51]. The existence of variation in how quickly embryos initiate ovary and testis differentiation in response to warm and cool temperatures, respectively, could be critical to allowing species with TSD to adapt to climate change.

We can study the molecular mechanisms underlying this variation in thermal responsiveness in *T. scripta* because the molecular sequence of events involved in TSD is well characterized [50,52–60]. At cool incubation temperatures, increased expression of histone demethylase (*Kdm6b*) appears to be responsible for demethylating the promoter of a critical regulator of testis differentiation, double-sex and mab−3-related transcription factor 1 (*Dmrt1*) [56]. However, when embryos experience even short exposures (24 h) to warm temperatures, *Kdm6b* expression decreases rapidly [54,57]. This response appears to be regulated by RNA splicing/intron retention, as the intron-containing transcript of *Kdm6b* (*Kdm6b*(+IR)) accumulates under cooler temperatures and rapidly declines following heat exposure [55,57]. From a mechanistic perspective, the rate at which the promoter of *Dmrt1* is demethylated may determine how quickly cool temperatures trigger the irreversible commitment to testis development, resulting in variation in thermal responsiveness. Conversely, *Cyp19A1* is a downstream gene of ovary development and encodes the aromatase enzyme responsible for converting androgens to oestrogens. Indeed, *Cyp19A1* is highly expressed upon ovary commitment, at which point the testis pathway cannot be induced perhaps in part owing to crosstalk between pathways [50]. Further studies also demonstrated that the stage of development when heat exposure occurs has strong effects on the response [52]. For example, experiencing a 14-day heat wave prior to day 17 of development or after day 38 of development in *T. scripta* produced primarily males, whereas a 14-day heat wave between those incubation days produced primarily females [52]. These studies collectively show how variation in the timing and duration of a heat wave exposure affects the response of sex determination in *T. scripta*. However, little to no work has focused on characterizing variation in the molecular responses to cool temperatures.

In this study, we examined the thermal responsiveness of testis development to cold snap exposures in *T. scripta* embryos. Specifically, we investigated how the timing and duration of cold snap exposure during embryonic development affect the expression of genes involved in gonadal development in *T. scripta*. Given that cool temperatures are testis-promoting in *T. scripta*, we chose to focus on the expression of an upstream testis-promoting gene (the intron-retaining transcript of the histone demethylase *Kdm6b*(+IR)), a downstream testis-promoting gene (the transcription factor *Dmrt1*) and a downstream ovary-promoting gene (*Cyp19A1*). Additionally, we examined how exposure duration from a cold snap starting early in development influences the proportion of resulting male hatchlings. We simulated cold snaps of different durations by shifting eggs from a baseline of female-producing temperatures (FPTs; 31 ± 3°C) to cooler, male-producing temperatures (MPTs; 26 ± 3°C) [51,52,61]. When maintained, these incubation temperatures produce approximately 100% females and 100% males, respectively [51–54]. We predicted that the expression of testis- and ovary-promoting genes in response to cold snap exposure would change rapidly (4–8 days) across embryonic development. Second, we predicted that the proportion of resulting male hatchlings would increase as the duration of a cold snap exposure increased, with a predicted proportion of 0% males under 0 day of exposure and approximately 100% males by 24 days of exposure.

## 2. Material and methods

### (a) Study system and egg incubation

To study the effects of cold snap timing and duration on the responsiveness of TSD, we utilized red-eared slider turtles (*Trachemys scripta*) as a model system. Eggs were collected on the day of oviposition from Concordia Turtle Farm, LLC (Jonesville, LA) and shipped to the laboratory at Illinois State University. We randomly assigned eggs across one of two studies and the treatments therein (control for clutch effects) before placing them into plastic boxes containing moist vermiculite. Although we used *T. scripta* eggs from a farm population in LA, a recent study suggests there is little variation in the sensitivity of sex determination between this farm population and a wild Illinois marsh population [62]. A separate study also suggests that three distinct and native North American populations of *T. scripta* (across IL, LA and SC) exhibit similar sensitivity in sex determination to heat wave exposures [51]. We therefore expect that this study’s results will be relevant to at least these wild populations but may have some limitations when extended to other populations or species.

In both experiments, eggs began incubation at a baseline of 31 ± 3°C corresponding to FPTs (IPP 110 Plus, Memmert GmbH+Co., KG, Schwabach, Germany). Cold snaps were simulated by moving eggs to incubators set at 26 ± 3°C, corresponding to MPTs. All incubators were programmed to hit their maximum at noon and minimum at midnight. The timing and duration of cold snap exposures depended on the assigned treatment group and sampling day. We chose these thermal conditions for two reasons: (i) the ±3°C fluctuations around these mean temperatures across several days have been observed in natural nests of *T. scripta* and (ii) when applied consistently across development, these FPT and MPT thermal regimes produce approximately 100% female and male hatchlings, respectively, meaning that each is capable of strongly inducing their respective gonadal pathways [51,52,61]. This latter point was further indicated by past gene expression work, which demonstrated that *Kdm6b* and *Dmrt1* reach higher levels in the gonads of *T. scripta* embryos maintained at 26 ± 3°C than those kept at 31 ± 3°C by about stage 17.5–18 (although there are no comparisons before stage 17 or beyond stage 21) [53]. Eggs were maintained at *ca* −150 kPa throughout incubation [51,52].

### (b) Experiment 1: the effect of cold snap timing on the responsiveness of genes involved in gonadal development

**Figure 1.**
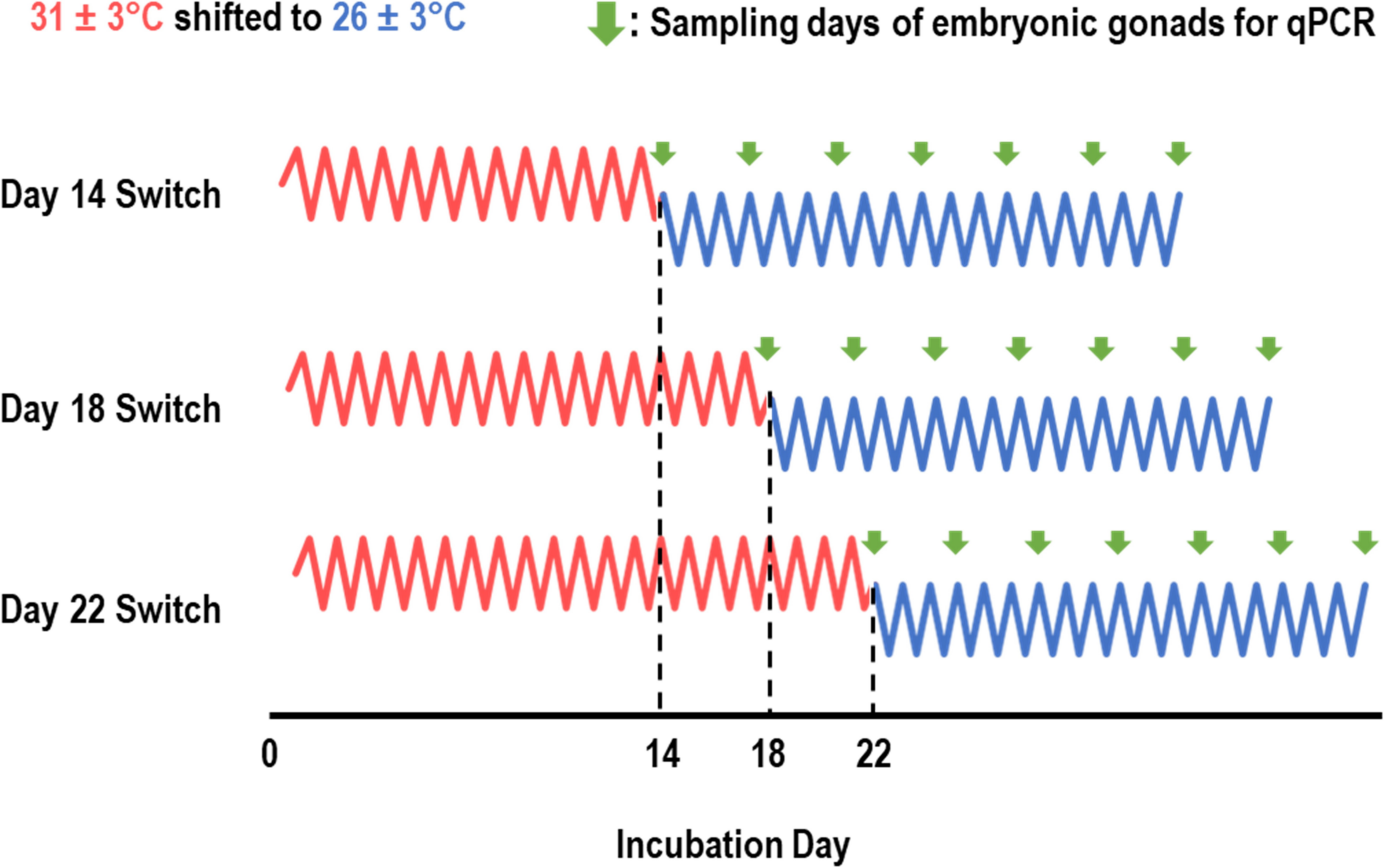
Incubation treatments and sampling points for experiment 1 exploring the effects of cold snap timing on the responsiveness of genes related to sex determination in *T. scripta* embryos. Thermal trace fluctuations are not to perfect scale.

Here, we sought to explore how the timing of cold snap exposure influences the responsiveness of genes associated with testis development (*Kdm6b*(+IR) and *Dmrt1*) and ovary development (*Cyp19A1*). To do this, we designed an experiment with three treatment groups that all began incubation at FPTs (31 ± 3°C) but varied regarding the timing of when eggs were shifted to MPTs (26 ± 3°C) to simulate a cold snap (figure 1). Eggs were shifted on either incubation day 14, 18 or 22. We then sampled gonads from subsets of embryos across each treatment just before the start of cold snap conditions (i.e. day 0) and on the 3rd, 6th, 9th, 12th, 15th and 18th days of cold snap exposure, roughly between 09:00 and 12:00 CDT on each sampling day. To ensure entire gonads were obtained from even the smallest embryos, we often collected some underlying adrenal and kidney tissues with each gonad. Individual samples corresponded to a specific embryo, treatment and sampling day and are therefore independent of other samples. Ultimately, 6–7 samples per sampling day per treatment were used for gene expression analysis. Samples were immediately placed into separate microcentrifuge tubes containing TRIzol Reagent (Ambion) and stored at −80°C until homogenization, from which RNA was extracted using 2-propanol (Fisher Chemical) and chloroform. Complementary DNA (cDNA) was synthesized from 1 μg RNA using the Maxima First Strand cDNA Synthesis Kit with dsDNase (Thermo Scientific), following the manufacturer’s instructions. These cDNA samples were diluted 1 : 10 using UltraPure^TM^ distilled water (Invitrogen) and loaded in triplicates on 384-well plates for real-time quantitative polymerase chain reaction (RT-qPCR) using PowerUp^TM^ SYBR^TM^ Green Master Mix (Applied Biosystems). This was performed using QuantStudio 7 Real-Time PCR System Software (Applied Biosystems) and the 2^−ΔΔCT^ method [63] was used to obtain the relative expression levels of *Kdm6b*(+IR), *Dmrt1* and *Cyp19A1*, each normalized to the sample’s expression of the house-keeping gene *Gapdh* [53,56,57]. Gene primers used in this study are published elsewhere: *Gapdh* [53], *Kdm6b*(+IR) [57], *Dmrt1* [64] and *Cyp19A1* [65].

### (c) Experiment 2: the effects of cold snap duration on sex ratios

**Figure 2.**
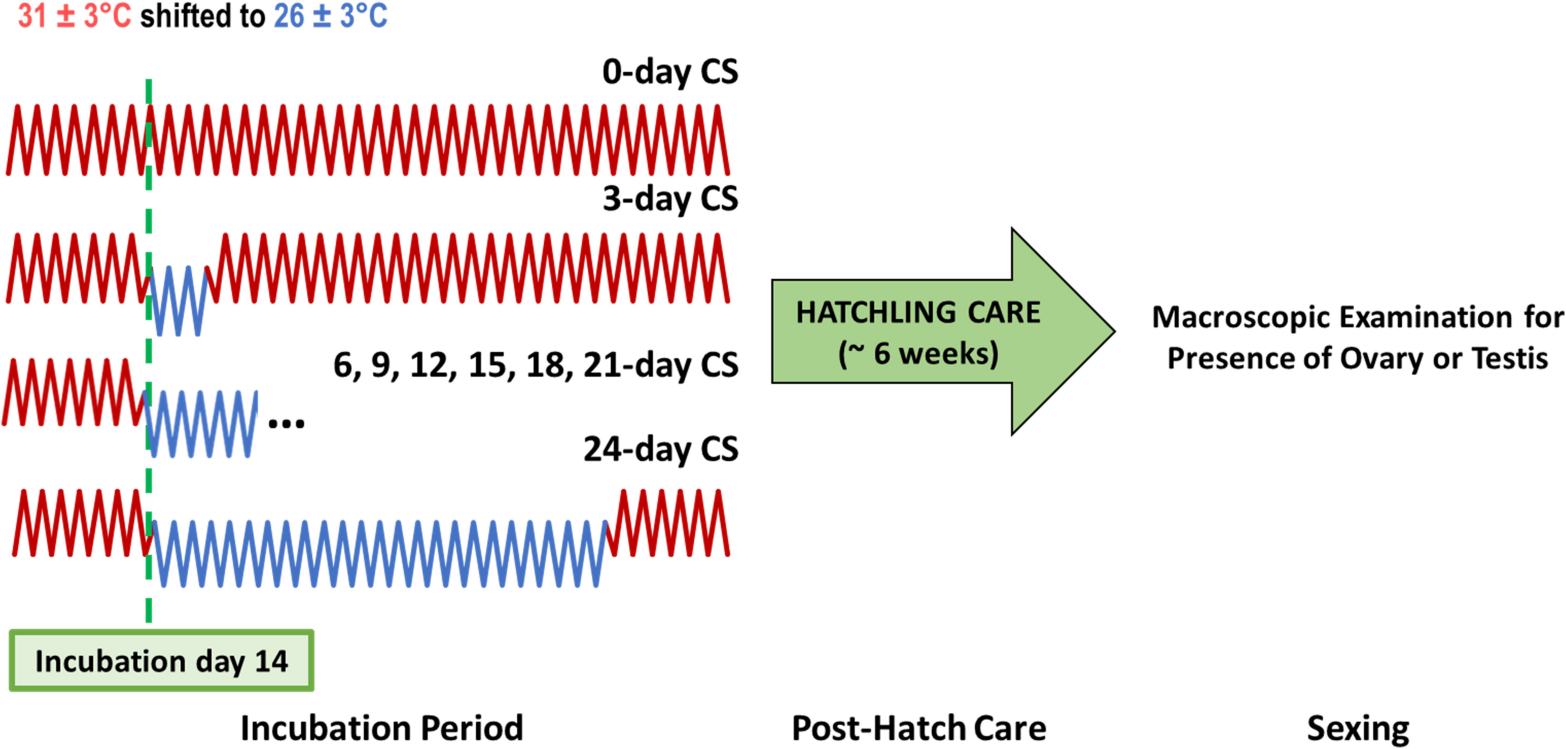
Experiment 2’s design to explore the effects of incubation cold snap (CS) duration on sex ratio outcomes in *T. scripta* hatchlings. Thermal trace fluctuations are not to perfect scale.

Here, we sought to characterize the effect of cold snap duration on resulting sex ratios. All eggs began incubation at 31 ± 3°C as in experiment 1. On incubation day 14, all treatments except the control (which remained at 31 ± 3°C for the duration of incubation) were shifted to cold snap conditions (26 ± 3°C), where they remained for either 3, 6, 9, 12, 15, 18, 21 or 24 days of exposure before returning to 31 ± 3°C until hatch (figure 2). We chose to introduce cold snap conditions here because previous studies indicated that incubation day 14, when developing under 31 ± 3°C, corresponds roughly to the start (stage 15) of the temperature-sensitive window (~stages 14–20) of gonadal development when sex is most responsive to temperature (although this window changes with the nature of incubation thermal conditions) [53,66]. Therefore, day 14 should represent roughly the start of the gonadal thermosensitive period and allow us to focus on the effects of exposure duration across that window. In addition, results from experiment 1 showed that *Dmrt1* steadily increased across 18 days of cold exposure beginning on day 14 (see §3 below), suggesting it was early enough to induce the testis pathway. We monitored for pipping (egg breach) daily following incubation day 65 [51]. On their day of pip, hatchlings and any remaining attached eggshells were transferred to individually labelled plastic cups and housed in the dark, at room temperature, on moist paper towels. Cups were cleaned with a dilute bleach solution every 5 days and fresh paper towels were provided until the hatchling’s residual yolk was fully absorbed. Afterwards, a shallow pool of fresh water was provided in place of paper towels. Investigators did not handle hatchlings apart from during water changes. Hatchlings were euthanized following approved methods at approximately 6 weeks post-hatch (ample time for oviduct regression in male hatchlings) and were subsequently sexed by visually inspecting the morphology of both gonads under a dissection scope [51,52,62]. The numbers of successfully developed (and thus sexed) hatchlings in each duration treatment (from 0 to 24 days of exposure) were 20, 15, 17, 17, 19, 20, 16, 18 and 19 hatchlings, respectively. All care and experimental treatment protocols involving hatchlings were in accordance with the Illinois State University’s Institutional Animal Care and Use Committee (IACUC).

### (d) Statistical analyses

Statistical analyses were performed in R (R Project for Statistical Computing, Vienna, Austria) [67]. To test the effect of cold snap timing on the expression of genes associated with gonadal development, a generalized linear model (GLM) was performed for each analysed gene specifying cold snap treatment (starting on day 14, 18 or 22), sampling point (1–7, representing 0, 3, 6, 9, 12, 15, and 18 days of cold snap exposure, respectively), and their interaction as fixed effects and a distribution that best met model assumptions. If GLMs produced significant effects of either cold snap treatment or an interaction between cold snap treatment and sampling point, *post hoc* comparisons were carried out only between the three treatments within each sampling point, and false discovery rate (FDR) adjustment was used to control for the probability of type 1 error. Only for models where treatment effects were non-significant and sampling point effects were significant did we run *post hoc* comparisons across sampling points by averaging across the three treatment groups at each day, again utilizing FDR adjustments.

The effect of cold snap exposure duration (*x*) on the proportion of males produced was estimated using a generalized log-logistic model with the *drc* package in R [68]. The best-fitting model had the following two-parameter log-logistic function [52]:

.$$f\left(x\right)=\;\frac{1}{\left(1+\exp\left(a+\log\left(x\right)-\log\left(b\right)\right)\right)}$$

This dose–response model is parameterized using a unified structure with a coefficient *a* denoting the steepness of the model (the Hill coefficient) and *b* denoting the effective dose producing 50% males (ED 50). This model parameterizes the upper and lower asymptotes of the response, which are constrained between 0 (0% males produced) and 1 (100% males produced).

## 3. Results

### (a) Cold snap timing and gene responsiveness

**Figure 3.**
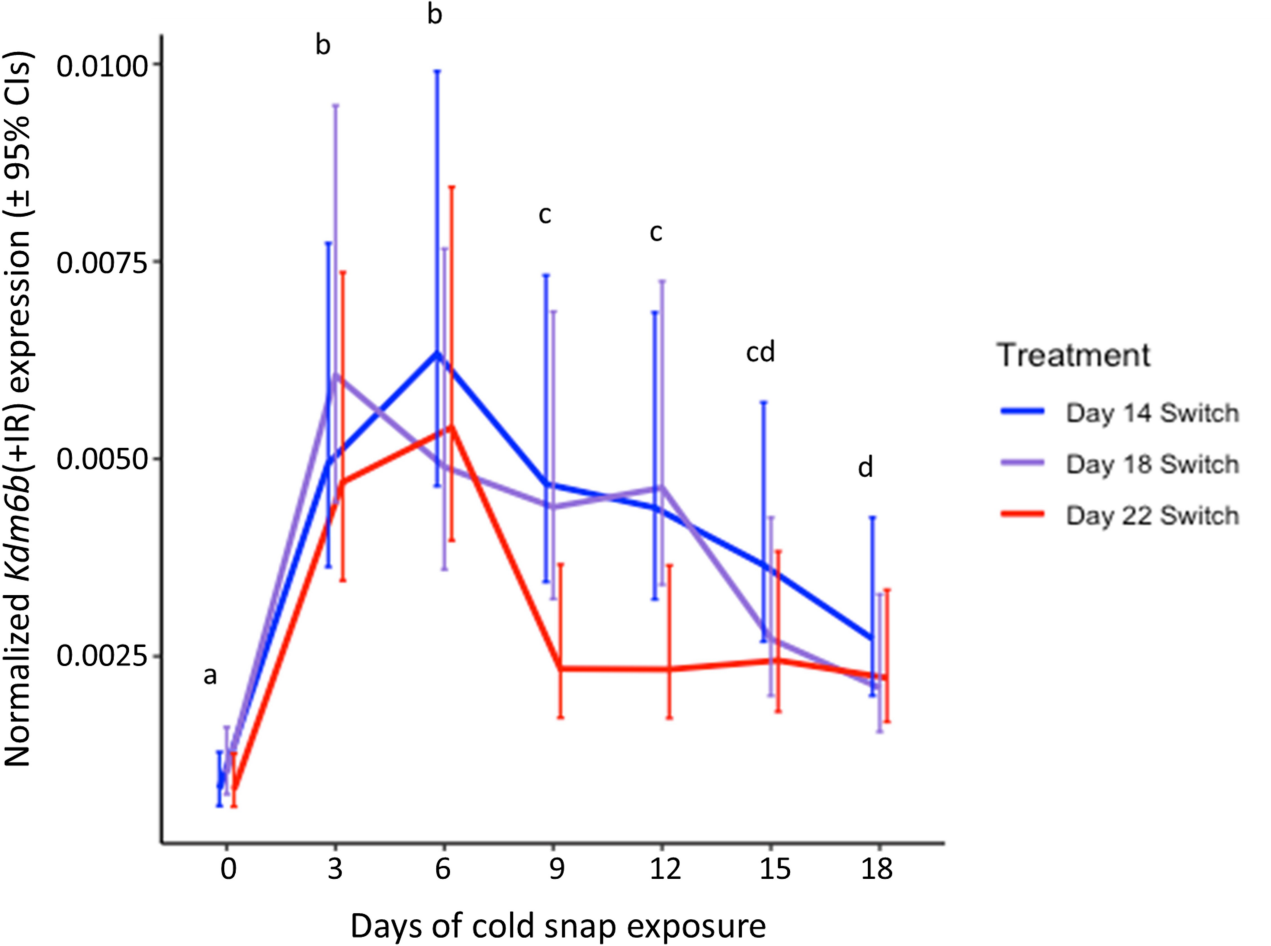
The estimated marginal means (±95% confidence intervals) of normalized *Kdm6b*(+IR) gene expression across the seven sampling points of an 18-day cold snap exposure for each cold snap induction treatment group. Significant differences between sampling points (days) averaged across the three treatments are indicated by letters above each day, where days not sharing a letter are significantly different.

**Figure 4.**
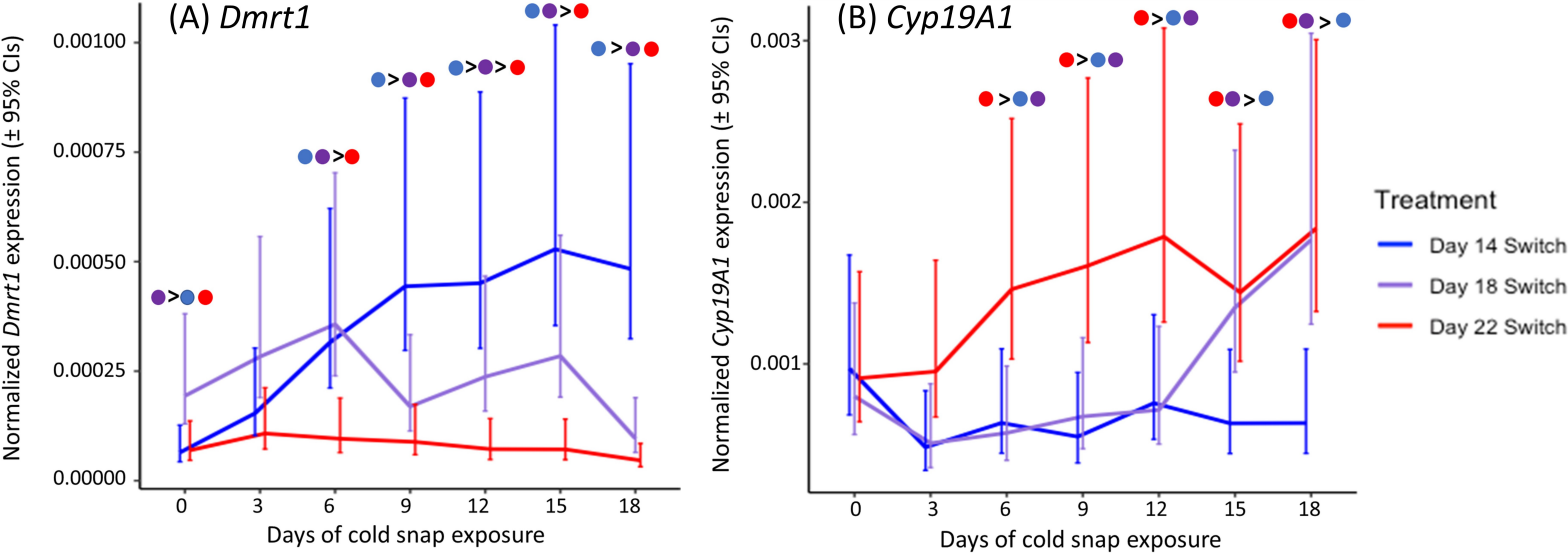
The estimated marginal means (±95% confidence intervals) of normalized *Dmrt1* (A) and *Cyp19A1* (B) gene expression across the seven sampling points of an 18-day cold snap exposure for each cold snap induction treatment group. Significant (*p* < 0.05) *post hoc* comparisons across treatments at each sampling point are indicated by circles coloured to match their respective treatments and those preceding a ‘>’ sign have higher expression than those following.

The embryonic response to cold snaps differed depending on which incubation day (and consequently stage) the cold snap was experienced. Embryos that experienced the cold snap starting on day 14 were at developmental stage 15, with embryos of the day 18 and day 22 cold snap treatments having development advanced by roughly 1–2 and 2–4 stages, respectively (table 1). For the upstream testis-promoting gene, *Kdm6b*(+IR), expression was rapidly induced by cold snap exposure and significantly changed across sampling points (*χ*^2^ = 167.57, *df* = 6, *p* < 0.0001), but this response was similar across treatments (*p* = 0.07; figure 3). For the downstream testis-promoting gene, *Dmrt1*, treatment (*χ*^2^ = 58.84, *df* = 2, *p* < 0.0001), sampling point (*χ*^2^ = 19.54, *df* = 6, *p* < 0.01) and their interaction (*χ*^2^ = 45.39, *df* = 12, *p* < 0.0001) were all significant. *Post-hoc* comparisons between treatments indicated that embryos experiencing the day 14 cold snap had an increased response compared to those not experiencing the exposure until day 22 of development (figure 4*a*). Meanwhile, the cold snap starting on day 18 saw an initial increase in *Dmrt1* expression similar to that of the day 14 treatment but this expression began to dissipate by day 9 (figure 4*a*). Lastly, for the downstream ovary-promoting gene, *Cyp19A1*, there was also a significant effect of treatment (*χ*^2^ = 44.75, *df* = 2, *p* < 0.0001), sampling point (*χ*^2^ = 15.08, *df* = 6, *p* < 0.05), and their interaction (*χ*^2^ = 25.96, *df* = 12, *p* < 0.05). Post-hoc comparisons showed divergence between treatments in *Cyp19A1* expression early in cold snap exposure, with expression in the day 22 treatment being significantly above the other two treatments. However, by the 15th day of exposure, the day 18 treatment group saw an increase in *Cyp19A1* expression to a level which matched that of the day 22 treatment group, whereas *Cyp19A1* expression remained low in the day 14 treatment group (figure 4*b*). Therefore, both *Dmrt1* and *Cyp19A1* expression diverged between the day 14 and day 22 treatments early in cold snap exposure and remained disparate across continued sampling points. Meanwhile, the day 18 treatment group began by closely matching the gene responses of the day 14 treatment but ultimately ended with expression patterns that instead more closely matched those of the day 22 treatment.

**Table 1.** Observed embryonic stages for each sampling point across the three cold snap treatments (day 14, day 18 and day 22).

sampling point	day 14 switch		day 18 switch		day 22 switch	
	**day**	**stage**	**day**	**stage**	**day**	**stage**
1	14	15	18	17	22	19
2	17	16	21	18	25	19.5
3	20	17	24	19	28	19.5
4	23	18	27	19.5	31	20
5	26	18.5	30	19.5–20	34	21
6	29	18.5–19	33	20	37	22
7	32	19	36	21	40	23

### (b) Cold snap duration and sex ratios

**Figure 5.**
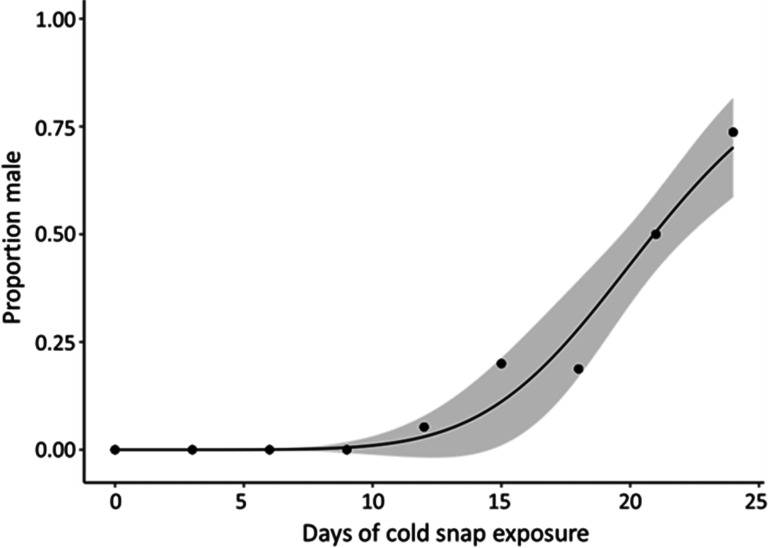
A duration-based reaction norm for the proportion of male hatchlings produced along increasing days of cold snap exposure during embryonic development in *T. scripta* as generated by a two-parameter log-logistic regression (+95% confidence bands). Cold snap exposures (shifted from 31 ± 3°C to 26 ± 3°C) began on incubation day 14 and were either 0, 3, 6, 9, 12, 15, 18, 21 or 24 days long.

The proportion of male hatchlings increased as the duration of cold snap exposure increased (*a* = −5.68 (s.e. = 1.22), *b* = 20.8 (s.e. = 1.0)). Specifically, the proportion of resulting male hatchlings remained at 0% (i.e. 100% females) across 0, 3, 6 and 9 days of cold snap exposure, then reached 5.3% males at 12 days of exposure, 20% males at 15 days, 18.75% males at 18 days, 50% males at 21 days of exposure, and finally 73.7% males at the maximum 24 days of exposure tested. The two-parameter log-logistic curve for the proportion of males produced across the 24 days of cold snap exposure is shown in figure 5.

## 4. Discussion

Climate change is likely to result in organisms experiencing transient exposures to unusual and extreme temperatures more frequently [1]. Most studies on climate change and TSD have focused on how organisms will respond to unusually warm temperatures, with fewer studies on how transient cool temperatures may similarly impact sex determination. Using *T. scripta* as a model, we investigated how cold snap timing and duration influence the response of key genes involved in testis and ovary development and resulting sex ratios. We found that embryonic responsiveness to cool temperatures changes rapidly across development in *T. scripta* and that early, prior exposures to warm temperatures are likely to affect this response. This was evidenced by the fact that a cold snap introduced on day 14 of development resulted in embryos expressing genes consistent with testis development, while embryos experiencing a cold snap just four to eight days later exhibited expression profiles consistent with ovary development. This suggests that on day 14 most embryos are still sensitive to an onset of cool MPT conditions, whereas the day 18 and 22 cold snap treatments occurred after the commitment to ovarian development was largely set in motion by the warm baseline FPTs. Moreover, embryos that remained at baseline FPTs for longer developed more quickly and thus later-timed cold snap treatments likely occurred later in (and lasted for a smaller proportion of) the temperature-sensitive window for sex determination [66]. Our sex ratio experiment also demonstrates that embryos experiencing a cold snap starting even on day 14 of development require a relatively long exposure (~21 days) under those cool temperatures before most (≥50%) individuals commit to testis development. Given this, it could be interesting to observe how downstream gene responses (e.g. *Dmrt1* and *Cyp19A1*) may vary among embryos of a day 14 cold snap treatment if embryos were then swapped back to FPTs after about 21 days into the exposure, because such variation could reflect whether individuals remain on trajectory for testis development or revert toward ovarian development. Indeed, we saw high variation among individuals in their *Dmrt1* response to cold conditions within the day 14 treatment alone (see figure 4*a*), and this variation in gene responsiveness may in turn reflect the variation in testis commitment observed from the sex ratio study. For example, we saw considerable variation in *Dmrt1* by the 18th day of exposure within the day 14 cold snap treatment, and in the sex ratio study, we see that only ~20% of turtles committed to testis development in response to 18 days at cold conditions beginning on day 14. It is therefore tempting to speculate that individuals that exhibit the quickest and greatest responses in *Dmrt1* (i.e. higher responsiveness) following the onset of cold exposure would also be those that are most likely to commit to testis formation. These results further highlight how individual gene responses to incubation temperature can change across embryonic development and vary considerably among individuals, both of which have potentially important implications for resulting sex ratios in nature.

If we consider how *T. scripta* embryos in natural nests might respond to altered thermal conditions, our findings suggest that variation in the timing of a cold snap can significantly impact how developing embryos will respond via the induction (or lack thereof) of the testis pathway. Our findings further indicate that, depending on the temperatures experienced prior to cold snap conditions, commitment to testis formation by most individuals depends heavily on the duration of exposure. We expect that the duration of cold exposure needed to induce testis commitment in most individuals would not need to be as long if the temperatures experienced before the exposure consisted of more male- or a mix of both male- and female-producing conditions, as might be the case for an early-season clutch in *T. scripta* (particularly if climate change results in advanced nesting). However, our experiments here may better model a late-season clutch experiencing a cold snap part way into their development. In this case, while transient cold exposure may be capable of inducing the expression of genes relating to testis development, our results suggest that it would need to be an unusually long exposure before most individuals would likely commit to testis formation, assuming those same individuals spent most of their prior development at FPTs.

Despite their large geographic distribution across diverse thermal environments, one study suggests that distinct *T. scripta* populations across three USA states (IL, LA and SC) have similar sensitivities to heat wave exposures in sex determination [51]. While we therefore expect that the sensitivity to cold snaps should be largely similar across these same *T. scripta* populations, more information is needed to determine any potential differences among various native and non-native populations, as has been suggested in other freshwater turtles [37]. In addition, maternally derived yolk estrogens, which promote ovary development and tend to be higher in late-season clutches in *T. scripta* [61], might further buffer developing turtles from unusual cold exposures (i.e. reduce responsiveness), but this has yet to be thoroughly explored [69]. When combined with the findings of earlier heat wave studies [51], our results suggest that developing *T. scripta* gonads are on average less likely to be affected by transient cool temperatures than developmentally comparable transient warm temperatures. This is evidenced by the *ca* 21 days of consistent cold exposure necessary to produce 50% males compared to just eight days of a developmentally comparable (stages 14–15) heat wave needed to produce 50% females [51]. The effect of heat waves may tend to be more potent on sex ratios than cold snaps because of an increase in developmental rate and/or a higher sensitivity in ovary-promoting genes relative to testis-promoting genes. However, whether an embryo exhibits low or high responsiveness to cool temperatures may still be crucial in determining gonadal fate under a more natural and thermally variable context. In fact, our findings demonstrate that some sex-determination genes (e.g. *Kdm6b* and *Dmrt1*) can respond to sudden cool temperatures late in development, even if the first 14–18 days consist solely of FPTs.

Interestingly, we found that *Kdm6b*(+IR) rapidly responded to cold snap exposure regardless of whether this began on day 14, 18 or 22, whereas the responsiveness of *Dmrt1* differed distinctly across timing treatments—from responsive (day 14) to practically non-responsive (day 22). Therefore, while *Dmrt1* is downstream of *Kdm6b* activity, its change in responsiveness across days 14 and 22 did not appear to correspond with any changes in the responsiveness of *Kdm6b*(+IR) during the exposure. Instead, this decrease in responsiveness of *Dmrt1* with later cold exposure may be owing to crosstalk from the ovary-promoting pathway arising earlier in development. For example, recent studies point to an influx of gonadal calcium and the induced phosphorylation of the signal transducer and activator of transcription 3 (pSTAT3) as a potential mechanism by which warm temperatures experienced early in *T. scripta* development establish a trajectory towards ovarian formation [58,70–72]. Recent studies suggest that early accumulation of pSTAT3 simultaneously inhibits *Kdm6b* transcription and promotes transcription of forkhead box l2 (*Foxl2*), an upstream transcription factor necessary for ovary development in *T. scripta* that may inhibit testis development by suppressing *Dmrt1* [70–72]. It is possible that our embryos that remained at baseline warm temperatures for longer exhibited greater accumulations of pSTAT3 early in development and thus higher *Foxl2* prior to the onset of cold snap conditions. If so, this could partially explain the decreased responsiveness of *Dmrt1* (owing to inhibition by *Foxl2*) despite no apparent changes in the responsiveness of *Kdm6b*(+IR). Similarly, an accumulation of *Foxl2* prior to cold snap treatment might explain the short-lived response of *Dmrt1* and subsequent increase in *Cyp19A1* seen in the intermediate day 18 cold snap treatment. It is also worth noting that *Kdm6b*(+IR) exhibited a sharper (albeit non-significant) decline after six days of exposure in the day 22 cold snap treatment relative to the day 14 and 18 cold snap treatments, which might be driven by alternative splicing and/or inhibition by pSTAT3 [54,57,72]. These mechanisms of crosstalk between sex-determining pathways are likely important components in determining the response of gonadal differentiation under naturalistic, fluctuating and transient temperatures and therefore warrant further study.

The time it takes for a response to occur under a given thermal cue during sexual development is clearly important when it comes to the induction of gonadal differentiation in TSD species. Yet, few studies to date have considered the time-course response of TSD to bouts of heat or cold. Variation in the responsiveness of genes involved in gonadal development to transient warm or cool temperatures may partially explain why sex ratios produced in nature can vary across (and sometimes within) clutches that experience fluctuations above and below the pivotal temperature [69,73,74]. Past work in painted turtles (*Chrysemys picta*) found that most (>60%) natural nests produce unisexual clutches [74], suggesting that variation in responsiveness is likely lower within clutches relative to across clutches. Similar responsiveness between clutch mates might be expected given their shared genetics and similar exposure to maternally derived yolk steroids. Such intraspecific variation in the inducibility of the testis and ovary pathways of TSD species may be relevant to their adaptive capacity in the face of rapid climate change, particularly if responsiveness is heritable.

Thermal responsiveness to cool temperatures in the nest may also be relevant to other physiological responses separate from TSD. For example, the induction of molecular chaperones, cryoprotectants and anaerobic energy pathways may shape the tolerance of hatchlings overwintering within the nest during cold snap exposures [75–78]. This might be important in turtle species like *T. scripta*, which often overwinter in their nest of origin as first-year hatchlings [79,80], and temperatures experienced during embryonic development might influence these responses later in life via the ‘programming’ of phenotype [81]. Warming resulting from anthropogenic climate change may also shorten incubation duration and thereby increase overwintering duration in hatchlings and/or alter the timing at which they experience cool temperatures [82], although it is not well known how potential phenological shifts might influence this. Therefore, in predicting the responses of oviparous species to climate change, it may be worth considering the thermal responsiveness of various pathways and life stages.

In conclusion, this study demonstrates how the sensitivity of molecular pathways underlying TSD (and the variation therein) can change rapidly across development in *T. scripta*. We speculate that variation among embryos in the thermal responsiveness of these molecular pathways may reflect the observed variation in gonadal commitment (sex ratios) in the context of naturalistic and transient incubation temperatures. However, relative to the time needed for ovary commitment to occur in most individuals in response to heat wave exposures [51], our results suggest that developmentally comparable cold snap exposures likely require considerably longer durations to induce testis commitment in most embryos. In other words, naturalistic heat waves might be expected to result in a relatively more potent effect on sex determination in *T. scripta* than naturalistic cold snaps, perhaps in part owing to their polarizing effects on developmental rate. Nonetheless, our study further highlights how the timing and duration of a transient thermal cue is important when considering their impact on sex determination and it extends this observation to include transient cold exposures. Further work is needed to determine the proximate mechanisms underlying changes in sensitivity across development (e.g. crosstalk between ovary and testis pathways) and its implications for the response of various TSD species to future climate change models.

## Data Availability

Data available from the Dryad Digital Repository [83].
